# Factors affecting the accuracy of a class prediction model in gene expression data

**DOI:** 10.1186/s12859-015-0610-4

**Published:** 2015-06-21

**Authors:** Putri W. Novianti, Victor L. Jong, Kit C. B. Roes, Marinus J. C. Eijkemans

**Affiliations:** 1 Biostatistics & Research Support, Julius Center for Health Sciences and Primary Care, University Medical Center Utrecht, 3508 GA Utrecht, The Netherlands; 2 Viroscience Lab, Erasmus Medical Center Rotterdam, 3015 CE Rotterdam, The Netherlands

## Abstract

**Background:**

Class prediction models have been shown to have varying performances in clinical gene expression datasets. Previous evaluation studies, mostly done in the field of cancer, showed that the accuracy of class prediction models differs from dataset to dataset and depends on the type of classification function. While a substantial amount of information is known about the characteristics of classification functions, little has been done to determine which characteristics of gene expression data have impact on the performance of a classifier. This study aims to empirically identify data characteristics that affect the predictive accuracy of classification models, outside of the field of cancer.

**Results:**

Datasets from twenty five studies meeting predefined inclusion and exclusion criteria were downloaded. Nine classification functions were chosen, falling within the categories: discriminant analyses or Bayes classifiers, tree based, regularization and shrinkage and nearest neighbors methods. Consequently, nine class prediction models were built for each dataset using the same procedure and their performances were evaluated by calculating their accuracies. The characteristics of each experiment were recorded, (i.e., observed disease, medical question, tissue/cell types and sample size) together with characteristics of the gene expression data, namely the number of differentially expressed genes, the fold changes and the within-class correlations. Their effects on the accuracy of a class prediction model were statistically assessed by random effects logistic regression. The number of differentially expressed genes and the average fold change had significant impact on the accuracy of a classification model and gave individual explained-variation in prediction accuracy of up to 72% and 57%, respectively. Multivariable random effects logistic regression with forward selection yielded the two aforementioned study factors and the within class correlation as factors affecting the accuracy of classification functions, explaining 91.5% of the between study variation.

**Conclusions:**

We evaluated study- and data-related factors that might explain the varying performances of classification functions in non-cancerous datasets. Our results showed that the number of differentially expressed genes, the fold change, and the correlation in gene expression data significantly affect the accuracy of class prediction models.

**Electronic supplementary material:**

The online version of this article (doi:10.1186/s12859-015-0610-4) contains supplementary material, which is available to authorized users.

## Background

As one of the major types of analyses for gene expression studies, supervised learning or classification has received high attention. Studies vary from the application of supervised methods to real-life problems like in [1–3], methods comparisons [4, 5] and methods development [6, 7]. Methods to build predictive models are widely available in the literature and it had been shown that the performance of a classification method varies, depending on the dataset to which the method is applied [8]. The characteristics of a dataset that naturally could be handled by a classification function might be one of the underlying reasons accounting for this variability. A classical method like linear discriminant analysis works under an assumption of the equality of covariance matrices between classes; while penalized logistic regression could handle a dataset with strongly correlated variables. Other specific study factors had also been shown to determine the predictive ability of a classification model, such as model building technique, array platform, clinical problem and sample size [9, 10]. Most of these characteristics are related to the technology or procedure and not to the specific data at hand. The characteristics of a gene expression dataset together with the nature of a classification function may play a key role in yielding a good class prediction model for gene expression data.

Evaluation studies on the aforementioned factors were based on classification models within the field of cancer. The effect of these factors might differ on datasets from non-cancerous diseases. This is because most cancerous diseases are often tissue specific unlike non-cancerous diseases that might involve the entire system and hence have different complexities. As one of gene expression data characteristics that has been proven by [11] to have an effect on the performance of probabilistic classifiers when calibration and refinement scores were used as model evaluation measurements, correlation structures have been shown to differ significantly between datasets from both cancerous and non-cancerous diseases [12]. These findings had led to the question, what factors do affect the performance of class prediction models on datasets from non-cancerous diseases. As such, a literature review study to quantify the association between study factors and the performance of classification methods outside the field of cancer was initiated [13]. The study, however, was limited to the characteristics of the microarray experiment, without investigating the effect of gene expression data characteristics such as the correlation between genes.

In this study, we outline potential study and data specific factors and assess their contribution to the accuracy of classification functions using real life gene expression data. The factors were chosen from both the experimental settings of the studies (i.e., disease, medical questions, tissue/cell types and sample size) and the characteristics of the gene expression data (i.e., the number of differentially expressed genes, the fold changes and the within-class correlations).

## Results

**Fig. 1:**
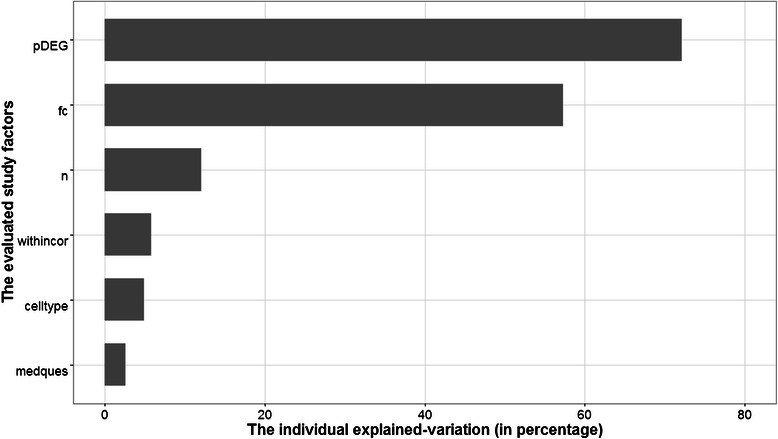
The individual explained-variation of study factors. Abbreviations: the number of differentially expressed genes on the log scale (pDEG), the fold change (fc), the sample size (n), the average within-class correlation coefficient (withincor), the cell type (celltype), and the medical question (medques)

On average, most classification methods performed better on hereditary disease. Meanwhile, the highest variability of the classification performance was observed on infectious disease (Additional file 4: Figure S1). Of the 25 experiments selected, 19 experiments addressed a diagnostic study. Diagnostic studies tend to be easily classified and hence yield higher accuracies than other (prognostic or response to a treatment) studies, as experienced by [14]. Despite this, the factor medical question is not significantly associated to accuracy (Additional file 5: Figure S2). A similar insignificant effect is also shown by cell type used in the experiment (Additional file 6: Figure S3). A more formal individual evaluation of the effect of each study factor to the predictive ability of a classification method was assessed by a random effects regression model as described in the Method section. The results of the individual evaluations are summarized in Table 1 and the individual explained-variation is depicted on Fig. 1.

**Table 1:** Individual random effect meta-regression

Study Factor	Coef*	AIC	P value	Individual explained-variation
Cell type	0.24 ^+^	137.9	0.44	4.87%
Medical question	−0.32 ^++^	137.8	0.38	2.55%
Sample size	−0.01	135.9	0.10	12.06%
The number of differentially expressed genes	0.21	116.0	<0.001	72.16%
Fold change	1.42	118.1	<0.001	57.31%
Within class correlation	1.74	137.5	0.31	5.80%

* Coefficient of the corresponding study factor in the random effects logistic regression

^+^ Coefficient for the *non-blood* category in the Cell Type study factor

^++^ Coefficient for the *non-diagnostic* category in the Medical Question study factor

The *fc* and *pDEG* study factor were positively associated to accuracy in their respective univariate random effects models. This intuitive finding confirms that a classification model could possibly achieve a good performance as the genes’ fold change or the number of differentially expressed genes increases (Additional file 7: Figure S4 and Additional file 8: S5). We transformed the *pDEG* to the log_2_-scale to deal with the high variability of the number of differentially expressed genes among studies, which ranged from 0 to 14,488.

Further, *pDEG* and *fc* had a relatively high individually explained-variation, i.e., 72% and 57%, respectively. Given its highest individual effect on the performance of classification model, we then used *pDEG* as the first factor entering the multiple regression model that was constructed by the forward selection approach. We stopped the modeling process when there was no additional study factor that improved the multiple regression model, conditional on the previously selected study factors in the model. The forward selection procedure yielded *pDEG*, *fc* and the within class average correlation (*withincor)* as the factors that simultaneously associated to the classification models accuracy. We referred this model as the final model of the multiple random effects logistic regression. The three study factors in the final model explained 91.5% of the random between study variation relative to the null model. As in the univariable case, *pDEG* and *fc* have positive effects on the accuracy of classification methods. Interestingly, *withincor* turned out to be one of the study factors that significantly improved the multiple regression model, although it was not significant univariately.

**Table 2:** Characteristic of the gene expression experiments

Disease	ID^+^	Medical question	Disease class	Cell/Tissue type	Affymetrix platform	Citation *	N	p	Ndeg	fc	cc
UC1	E-GEOD-14580	Response to treatment (non-/responder)	Inflammation	Colonic mucosal biopsies	HG U133 Plus 2.0	yes	24 (16,8)	4650	623	1.551	0.162
UC2	E-GEOD-21231	Response to treatment (non-/responder)	Inflammation	Blood	HG 1.0 ST	yes	40 (20,20)	3388	0	0.207	0.112
UC3	E-GEOD-36807	Diagnostic (UC/CD)	Inflammation	Intestinal biopsy	HG U133 Plus 2.0	no	28 (15,13)	6541	21	2.222	0.305
UC4	E-GEOD-23597	Response to treatment (non-/responder)	Inflammation	Colonic biopsy	HG U133 Plus 2.0	yes	14 (7,7)	4793	0	1.119	0.298
UC5	E-MTAB-331	Diagnostic (UC/CD)	Inflammation	CD8+ T cell	HG 1.0 ST and HG 1.1 ST	yes	59 (30,29)	1402	312	0.714	0.164
UC6	E-GEOD-9452	Diagnostic (with/without inflammation)	Inflammation	Colon	HG U133 Plus 2.0	yes	17 (8,9)	3702	2401	3.697	0.165
UC7	E-GEOD-6731	Diagnostic (UC/CD)	Inflammation	Colon	HG U95AV2	yes	30 (11,19)	1055	0	0.485	0.228
AST1	E-GEOD-27011	Diagnostic (mild/severe)	Inflammation	Blood	HG 1.0 ST	no	36 (19,17)	1293	39	0.302	0.113
AST2	E-GEOD-51392	Diagnostic (asthma/rhinitis)	Inflammation	Bronchial epithelial cells	HG U133 Plus 2.0	no	11 (6,5)	3969	0	1.805	0.171
AST3	E-GEOD-31773	Diagnostic (non/severe)	Inflammation	CD4 T cells	HG U133 Plus 2.0	no	12 (4,8)	18321	14488	16.964	0.317
DYS	E-GEOD-19419	Diagnosis (carrier/symp)	Infection	Blood	HG 1.0 ST	yes	45 (22,23)	2811	0	0.182	0.153
HIV1	E-GEOD-35864	Diagnostic (HIV/HIV with complication)	Infection	Basal ganglia	HG U133 Plus 2.0	no	18(6,12)	8737	0	1.14	0.346
HIV2	E-GEOD-14278	Prognostic (resistant/susceptible)	Infection	Peripheral blood	HG U133 Plus 2.0	no	18 (9,9)	11286	4	0.58	0.12
HIV3	E-GEOD-6740	Diagnostic (chronic/non chronic)	Infection	CD4 T cell	HG U133A	yes	15 (10,5)	865	5	0.74	0.168
PSO	E-GEOD-18948	Response to treatment (non-/responder)	Immune	Blood	HG U95	yes	16 (7,9)	1987	34	1.131	0.369
KD	E-GEOD-16797	Response to treatment (IVIG responsive /non)	Immune	Blood	HG U133 Plus 2.0	yes	12 (6,6)	11043	5	1.688	0.224
Dia1	E-GEOD-18732	Diagnostic (type 2 diabetes/intolerant)	Immune	Skeletal muscle	HG U133 Plus 2.0	no	71 (45,26)	2038	10	0.279	0.16
Dia2	E-CBIL-30	Diagnostic (diabetes type 2/abnormal glucose)	Immune	Skeletal muscle	HG U133A	yes	26 (18,8)	1749	0	0.269	0.435
ALZ1	E-GEOD-1297	Diagnostic (severe/not severe)	Degenerative	Hippocampus	HG U133A	yes	22 (7;15)	2295	13	0.693	0.287
ALZ2	E-MEXP-2280	Diagnostic (Alz/Pick's disease)	Degenerative	Medial temporal lobe	HG U133 Plus 2.0	yes	19 (7,12)	6899	1592	1.086	0.231
PARKI	E-GEOD-6613	Diagnostic (Parkinson/non-Parkinson)	Degenerative	Blood	HG U133A	yes	83 (50,33)	638	0	0.192	0.361
HF	E-GEOD-26887	Diagnostic (with/-out Diabetes)	Degenerative	Left ventricle cardiac biopsies	HG 1.0 ST	yes	19 (7,12)	2068	0	0.374	0.131
GAU	E-GEOD-21899	Diagnostic (type 1/ 3)	Hereditary	Skin	HG U133A 2.0	no	10 (5,5)	2017	4	1.807	0.143
CS	E-MEXP-2236	Diagnostic (Apert/Muenke)	Hereditary	Skin	HG U133 Plus 2.0	yes	20 (10;10)	5422	21	0.59	0.255
CF	E-GEOD-10406	Diagnostic (Chronic rhinosinusitis/+Cystic fibrosis)	Hereditary	Sinus mucosa	HG U133 Plus 2.0	no	15 (9,6)	7604	0	0.786	0.206

+ : The ArrayExpress accessing ID

* : Paper availability

Ndeg : The number of differentially expressed probesets

fc : The average fold change from all probesets

cc : The average within class correlation values from all probesets

Despite a relatively small number of studies, the random effects logistic regression model was stable, as shown by the high agreement of the random effects logistic regression models in the Jackknife resampling analyses. The Jackknife analysis was done by leaving out one study at a time and rebuilding the random effects regression model in the remaining studies. In the univariable evaluation of Jacknife resampling, the *fc* and *pDEG* study factors were always found to be significant in the random effects models. The *sample size*, however, came as one of significant study factors five times, i.e., when *uc4*, *uc5*, *hiv3*, *kd* and *hf* studies were left out from the random effect models (Additional file 2: Table S1). In the multivariable evaluation, the significant study factors in the final model were selected 19 times out of 25 Jackknife samples yielding a robustness of 76%. The *pDEG*, *withincor,* and *fc* were in the model for 25 times (100 %), 24 times (96 %) and 19 times (76 %), respectively (Additional file 3: Table S2).

## Discussion

We enumerated possible characteristics of gene expression data and investigated their impact on the predictive accuracy of nine chosen classification methods using twenty-five downloaded gene expression datasets. While a substantial amount of information is known about the characteristics of classification methods, little has been done to determine which characteristics of gene expression data affect the performance of a classifier. Classification methods have been shown to have varying performances in gene expression datasets. The classification methods, on average, performed differently across the different disease types (Additional file 4: Figure S1), but the random effects logistic regression model failed to show a significant relationship between disease type and the accuracy of classification models. This might be as a result of the limited number of samples available to evaluate such a factor with five categories.

In general, we might have an issue of statistical power and model over-fitting when considering this variable. A solution could be to increase the number of studies by adding cancer studies to increase the statistical power and possibly lead to a comparison in different behavior of the study factors between cancerous and non-cancerous diseases. However, supervised learning on gene expression studies in the field of cancer have been studied extensively by [9, 10, 15]. As such, we chose to focus on microarray gene expression experiments outside the field of cancer. We assessed the stability of the results from both univariable and multivariable random effects logistic regression via Jackknife resampling. We excluded one dataset for each sampling and repeated the random effects modeling process. We then recorded P values of each study factor in univariable models and the study factors that were included in the model in multivariable evaluation. Large number of datasets needs to be included in order to yield more generalizable results and also to avoid underpowered findings, particularly in an evaluation or comparison study [16]. Nevertheless, the evaluation of our results by Jackknife resampling shows high stability of our results and high agreement as compared to the findings by using full datasets

A similar study that was based on a quantitative review was conducted to evaluate study factors that were associated with the performance of classification models in the non-cancer field [13]. That study had found that the cross-validation technique considerably affected the predictive ability of classification models, in line with the finding of MAQC II consortium study [9]. In the current study, we then controlled for the effect of cross-validation technique to observe the effect of other study factors that could not be observed earlier in [13]. The same predictive modeling technique, including cross-validation, feature selection and classification functions, was applied to the preprocessed gene expression datasets. The performance of the optimum classification models were measured by calculating the proportion of correctly classified samples and total sample size. Random effects logistic regression models showed that gene expression data characteristics such as fold changes, the number of differentially expressed genes and the correlation between genes, contribute to the performance of classification models.

We used classification accuracy as the outcome of analysis. Although it is well-known to be a rough measure for the performance of a classification model, accuracy is widely used in practice due to its straightforward interpretation. In highly imbalanced datasets, accuracy may yield overoptimistic results, because a classification model might easily send all samples to the majority class. The class imbalance should therefore be taken into account when interpreting prediction accuracy [15]. A meaningful classification model necessarily should have higher accuracy than the proportion of the majority class. To deal with the problem of class imbalance when using accuracy, we corrected our random effects models for the class imbalance level.

We showed that the number of the differentially expressed genes, genes’ fold changes and the average within-class pairwise correlations significantly affected the accuracy of classification models. The positive coefficient of the number of differentially expressed genes (*pDEG*) both in the simple and multiple random effects models shows that the classification models performed better if the number of differentially expressed genes present in a dataset is increased. Similarly, fold change (*fc*) was significant in both univariable and multivariable evaluations with positive effects. These intuitive findings were mentioned earlier by the MAQC II consortium study [9], where the number of informative genes had relatively high degree of explained variability of the classification model performance in cancer studies.

**Fig. 2:**
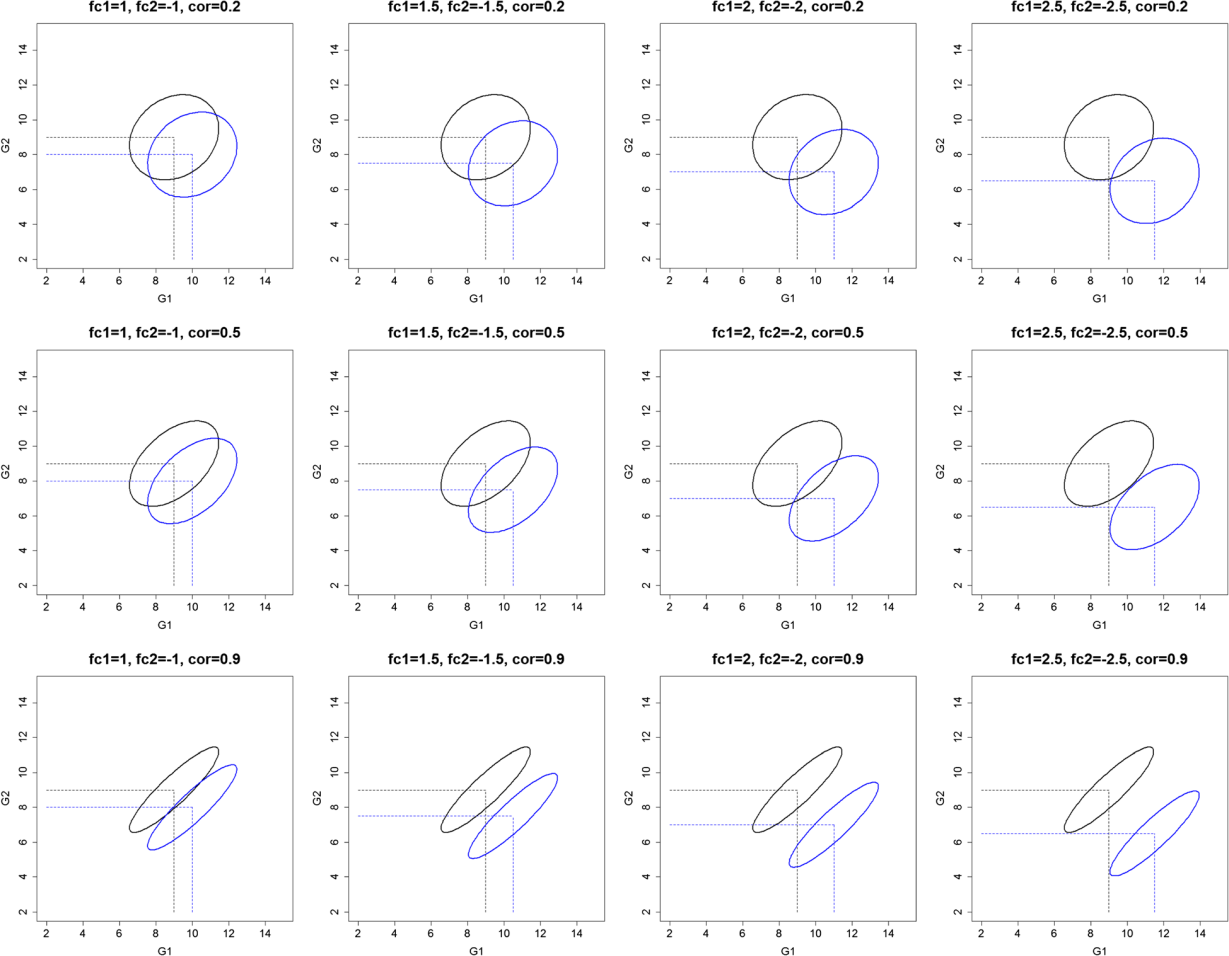
Visualization of the generated gene expression datasets with the scenario of fc1 = +,fc2 = −,cc1 = cc2 = +. Abbreviations: fc1(2): fold change of gene 1 (2); cc1(2): correlation coefficient of gene 1 (2)

The within-class correlation (*withincor*) has a positive effect on the accuracy of classification models together with *pDEG* and *fc* in the final random effect model. The positive effect of the *withincor* study factor to the classification model performance, is in contrast to knowledge from linear models that correlated variables bring no additional information to the model and therefore tend to reduce the predictive ability of the model. Our results show that the relationship between *withincor* and model accuracy is confounded by the *pDEG* and *fc.* To explain this finding, let’s first consider the within class correlation between two genes, both with a certain fold change. The two classes are more separable when the pairwise within class correlation between two genes becomes stronger (Fig. 2: one gene up- and the other down regulated and positive within class correlation and S7: both genes up regulated and negative within class correlation). Meanwhile, we hardly observe an effect of the within class correlation in the other possible scenarios (Additional file 9: Figure S8: one gene up- and the other down regulated and negative within class correlation and S9: both genes up regulated and positive within class correlation). Thus, there are two possible effects of the within class correlation to the classification model‘s performance, i.e., either positive or no effect, which might be the reason for an overall significant positive coefficient of the *withincor* study factor.

The theoretical examples given above concern probesets with relatively high fold changes, reflecting the probesets that were involved in the classification models. In our classification approach, we ranked probesets based on the limma feature selection methods and used top-K probesets to feed the classifiers, as commonly done in practice, e.g., by [2, 3, 17–20] in non-cancer and [14] in the cancerous diseases. By using this approach, we ensured that the probesets involved in the classification models had considerable fold changes. Thus, it supports the confounding effect of the *fc* study factor to the *withincor* in the multivariable random effect regression model.

The correlation structure in gene expression data had been proven to have a negative impact on the performance of probabilistic classifiers [11]. This could possibly be due to the measure of evaluation and/or the fact that all genes were used and not a top number from a ranked list. In the non-probabilistic classifier, its effect has not been studied yet. The result of this study could be a preliminary proof of the effect of correlation between genes (or probesets) to the performance of general classification models (for both probabilistic and non-probabilistic classifiers). Given our results, a similar simulation study as [11] by considering broad range of combination values of fold changes, the number of informative genes and correlation structure of a gene expression dataset, is worth initiating by applying both probabilistic and non-probabilistic functions.

## Conclusions

We evaluated factors that possibly had an impact on the performance of classification models in gene expression experiments outside the field of cancer. The factors were categorized into two main groups: the study- and the data-related factors. Our study showed that the data-related factors ‘number of differentially expressed genes’, ‘fold change’, and ‘within-class correlation’ significantly affect the accuracy of classification functions.

## Methods

### Data extraction

We downloaded microarray gene expression datasets from the ArrayExpress data repository. The criteria for selecting the datasets were that the experiments 1) had been conducted in humans; 2) outside the field of cancer; 3) had samples with class labels in at least two classes; 4) were published after 2005; and 5) provided raw cell files. To reduce the source of variability of classification model performances because of the array used in the experiments, we retained studies conducted with the only Affymetrix array. This additional exclusion criterion was also motivated by the widely used of Affymetrix array by studies that were recorded in the ArrayExpress repository. Out of 54169 recorded studies in the ArrayExpress, 21284 (39.2%), 4436 (8.2%) and 3896 (7.2 %) studies used Affymetrix, Illumina and Agilent array, respectively (last checked in November 18, 2014). We took only two disease classes or dichotomized the outcomes if there were more than two classes in a study. In total, we downloaded twenty five gene expression datasets [2, 17, 21–34] briefly described in the Supplementary Material (Additional file 1) and summarized in Table 2. In addition to the extracted datasets, the following study characteristics were recorded: medical question addressed, disease type, tissue/cell type, microarray platform, paper availability, year of publication and sample size. The twenty five gene expression datasets came from microarray studies that were conducted in thirteen different diseases. We grouped the diseases based on etiology resulting in five major types namely; inflammatory (10), infectious (4), immune (4), degenerative (4), and hereditary (3) diseases. The disease grouping was aimed to evaluate the potential effect of the disease complexity to the performance of the classification methods.

### Preprocessing

The raw datasets were normalized using quantile normalization, background correction performed according to manufacturer’s platform recommended correction and log base two transformed [28]. Median polish was used as a summarization method to quantify expression values because of its ability to deal with outlying probes [29]. For each dataset, we filtered out non-informative probesets using two filtering criteria. First, we retained probesets that had expression values greater than five in at least ten percent (10%) of the total samples. Secondly, we filtered the retained probesets whose standard deviations were greater than 0.5 (sd > 0.5). We refer to the retained list as the actual expression data.

### Classifier building

We built and evaluated in each dataset class prediction models with the set of nine classifiers described in the classification functions subsection. Since we are only equipped with a finite sample and the underlying distribution is unknown, the empirical counterpart to the generalization accuracy of a classification function *f* is estimated as

1 $$ {R}_{emp}\left[f\right]=\frac{1}{n}{\displaystyle \sum_{i=1}^n}L\left({y}_i,f\left({x}_i\right)\right), $$

where *n* is the number of available samples and *L*(.,.) is a loss function with *L*(*u*, *v*) = 1 if *u* = *v, L*(*u*, *v*) = 0 otherwise [35].

Though this empirical counterpart to the generalization accuracy can be used to evaluate classifiers, it usually overfits the sample $\mathcal{S}$ *.* A general practice is to split the samples into a learning set ℒ and a testing set $\mathcal{T}$. Predicted value from a classification function $\hat{f}(.)$ is constructed from a learning set ℒ only and evaluated using a testing set $\mathcal{T}$ [35]. In case sample sizes are very small, a good practice is to generate several learning and testing sets from the available sample, construct a classifier with each learning set and using the corresponding testing set, estimate the empirical generalization accuracy. The final empirical generalization accuracy is the average across the testing sets. Suppose *B* learning sets ℒ_*b*_ (*b* = 1, … *B*) are generated from sample $\mathcal{S}$ and the corresponding testing sets $\mathcal{T}=\mathcal{S}\backslash {\mathrm{\mathcal{L}}}_b$ with ${\hat{f}}_b(.)$ obtained from ℒ_*b*_ (*b* = 1, … *B*) then an estimate of the accuracy is calculated by

2 $$ acc=\frac{1}{B}{\displaystyle \sum_{b=1}^B}\frac{1}{\left|{\mathcal{T}}_b\right|}{\displaystyle \sum_{i\epsilon {T}_b}}L\left({y}_i\hat{f_b}\left({x}_i\right)\right), $$

where | ⋅ | is the cardinality of the considered set [35].

As such, each dataset was split into two-thirds for the learning set and one-third for the testing set taking into account the number of samples per class (i.e., stratified sampling), using Monte Carlo cross-validation (MCCV) [35] and the probesets were ranked using the moderated t-statistic [36] on the learning set. The learning set was further split into an inner-learning set and an inner-testing set using leave one out cross-validation (LOOCV). The parameter(s) of the classification functions (if any) were tuned by ranking the probesets on the moderated t-statistic and building the classifier with different values of the parameter(s) using the inner-learning set and evaluated with the out of bag inner-testing set as proposed by [37]. The number of top probesets to be included in the classification function was also determined among *p* = 5, 10, 15, 20, 25, 50, 55 for non-discriminant and *p* = 2, 3, 4, 5 (except for the GAU dataset, *p* = 2, 3) for linear discriminant analysis (LDA) and diagonal linear discriminant analysis (DLDA) using the corresponding inner-learning and inner-testing sets. The restriction of the top probesets for the discriminant functions is due to the inability of these functions to accommodate a number of probesets greater than the number of samples. With the optimal probeset(s) and number of top probesets (*p*) for each classification function, the class prediction model was built for each classification function using the learning set and then evaluated within the testing set. The process was repeated *B* = 100 times. The numbers of correctly-classified and misclassified samples in both learning and testing sets were then recorded.

### Classification functions

The nine classification functions were chosen to represent the broad list in the literature that falls within the categories: discriminant analyses or Bayesian (linear discriminant analysis (LDA), diagonal linear discriminant analysis (DLDA), and shrunken centroid discriminant analysis (SCDA)), tree base (random forest (RF) and tree-based boosting (TBB)), regularization and shrinkage (RIDGE, LASSO and support vector machines (SVM)), and *k*-nearest neighbors (*k*NN) methods *K*-nearest neighbour (*K*NN).

#### Linear discriminant analysis (LDA)

Discriminant analyses are Bayes optimal classifiers, which assume that the conditional distributions of predictors given the classes are multivariate normally distributed and the within-class covariance matrices are equal for all classes [35]. In order to get an optimum LDA classifier, we optimized the number of probesets to be included in the model.

#### Diagonal linear discriminant analysis (DLDA)

As LDA, DLDA also works under the assumption of multivariate normality of class densities and a diagonal within-class covariance matrix for each class [35]. The optimum number of probesets was tuned by cross-validation.

#### Shrunken centroid discriminant analysis (SCDA)

It is also well-known as the prediction analysis of microarray (PAM) and it is specially developed to handle the high-dimensionality of gene expression microarray data. The method works by shrinking the class centroids to the overall centroid. For binary classification, the mean for each probesest *j* in each class *k* is calculated, and is called the class centroid. The class centroids are first normalized by overall mean, pooled standard deviation and sample sizeThis normalized class centroid is denoted by *d*_*jk*_. The goal of this method is to shrink *d*_*jk*_ towards zero by reducing *d*_*jk*_ by an amount of *Λ*. A large *Λ* value implicitly means excluding more probesets, which lead to a reduction in the model complexity. On the other hand, less number of probesets in a model would increase the risk of excluding informative probesets [38]. To balance this trade-off, parameter *Λ* was optimized amongst the following values: 0.1, 0.25, 0.5, 1, 2, and 5. SCDA is categorized as an embedded filtering method because of its ability to do filtering and model building simultaneously [39].

#### Random forest (RF)

Random forest is a classification method designed for decision tree classifiers. It combines the predictions made by multiple decision trees to yield the final prediction of a test sample. Supposed the sample size of the training set is *N*, each tree is constructed by: (i) sampling with replacement a random sample of cases of size $\frac{2}{3}N$ and (ii) at each node, a random sample of predictor variables *m* sampled from all predictor variables is selected and the predictor variable with the best split based on a given objective function is used. Step (ii) above is repeated until the tree is grown to terminal nodes with minimum size *k*. The out-of-bag (oob) samples are used to evaluate the constructed tree. Randomization helps to reduce the correlations among decision trees so that the generalization accuracy of the classifier can be improved. A higher value for the minimum terminal node size *k* would possibly lead to smaller grown trees. Once multiple trees have been built, they are then combined by voting; that is each tree cast a vote at its terminal nodes [40]. The parameters *m* and *k* are often optimized using cross-validation. In this study, we fixed the number of trees in a forest at 500 and the number of random probesets at each split *m* and the minimum terminal nodes size *k* were tuned within the values $\left(\left(0.1,\ 0.25,\ 0.5,\ 1,\ 2\right)*\sqrt{p}\right)$ and (1, 2, 3), respectively. Where *p* is the total number of probesets.

#### Tree-based boosting (TBB)

Boosting is a classification method that combines the output of several “weak” classifiers to produce a powerful “committee” [41]. It is an iterative procedure used to adaptively change the distribution of the training samples so that the base classifiers focus on samples that are hard to classify. Boosting assigns a weight to each learning sample and may adaptively change the weight at the end of each boosting round. These weights are then used either as a sampling distribution or can be used by the base classifier to learn a model that is biased toward higher-weight samples. The idea is to give all observations the same weights at the start, draw a bootstrap sample and build a classifier, which in this case is a classification tree (hence tree-based boosting) then test the classifier with all the subjects. The weights of misclassified subjects are increased in the next bootstrap sample thereby given them higher chances to be sampled. We optimized the number of trees (bootstrap samples) that falls within these following values: 50, 100, 200, 500 and 1000.

#### Ridge regression (RIDGE)

The L2-penalization is used in logistic regression to shrink the less significant coefficients toward zero. The amount of shrinkage is controlled by a parameter *λ*, where larger *λ* implies a larger degree of shrinkage [41]. The parameter *λ* of the penalization is a tuning parameter obtained by cross-validation (*λ* = 0.0625, 0.125, 0.25,0.5, 1, 2, 4, 8, and 16).

#### LASSO

As in ridge regression, LASSO uses a penalization parameter (*λ*) to estimate the coefficients of logistic regression, this time using L1-penalization. *λ* is interpreted as truncating the less significant coefficients, so that LASSO also works as a method for variable selection. We selected the optimum *λ* parameter within the range 0.1:0.9 by 0.1 using cross-validation [41].

#### Support vector machines (SVM)

SVM classification is a binary classification method that fits an optimal hyperplane between two classes by maximizing the margin between the classes' closest points. The points lying on the boundaries are called support vectors, and the middle of the margin is the optimal separating hyperplane. Data points on the “wrong” side of the discriminant margin are weighted down to reduce their influence and it is controlled by the cost parameter *C*. For the nonlinear case, SVM uses a nonlinear mapping (via kernels) to transform the original training data into a higher dimension. Within this new dimension, it searches for the linear optimal separating hyperplane that is, a “decision boundary” separating the tuples of one class from another. The SVM finds this hyperplane using the support vectors (“essential” training tuples) and margins are defined by the support vectors [42]. We used a linear kernel and the optimal cost parameter was obtained from 0.1, 1, 5, 10, 50, 100, 500 using cross-validation.

#### K-nearest neighbor (KNN)

For a sample $\mathcal{S}$, the *K*NN algorithm classifies this sample $\mathcal{S}$ based on a measure of distance between $\mathcal{S}$ and other learning samples. It finds the *K* samples in the learning set closest to $\mathcal{S}$ and then predicts the class of . by majority votes. The value *K* is usually specified by the user. It should be noted that if *K* is too small, then the nearest-neighbor classifier may be susceptible to over-fitting. On the other hand, if *K* is too large, the nearest-neighbor classifier may misclassify the test instance, because its list of nearest neighbors may include data that are located far away from its neighborhood [43]. The optimal value of *K* is chosen by cross-validating amongst *K* = 1 : 10 by 1.

## Predictive factors

The study characteristics (referred to as “study factors”) were evaluated for their effect on the performance of the classification methods. The factors were chosen from both the experimental settings of the studies and the characteristics of the gene expression data. We selected study factors that have been proven in the literature or intuitively have association with the performance of classification models. To represent the experimental setting, we chose study factors like medical question, sample size and cell/tissue type used in the experiment. The gene expression data were explored further to find the characteristics that might contribute to the performance of classification methods, namely the number of differentially expressed genes, fold changes and within-class pairwise correlations. The study factors are described as follows:

### *Medical question*

The medical questions were of different types: diagnostic, prognostic and response to treatment related studies. Diagnostic studies tend to have higher classification model performance than prognostic or response to a treatment studies, as experienced by e.g., [14]. This factor also came out as one of the factors that was associated with classification model performance outside the field of cancer [13]. We classified the medical questions of the experiments as either diagnostic or non-diagnostic.

### *Sample size*

Microarray datasets suffer from a severe curse of dimensionality. The impact of the number of samples used in the analysis was therefore investigated, particularly in the field of cancer by [10] . The class imbalance is another point of consideration when building a classification model. It may introduce bias towards the majority class in a prediction model and the classification performance will be overestimated, especially when the accuracy is used to evaluate the model [44]. The class imbalance factor is calculated as the number of samples in the majority class divided by the total sample size.

### *Cell type*

The tissue or cell type used in the experiment is likely to be dissimilar between studies and may impact the resolution of information and also the performance of classifiers. In a specific cancer case, like in acute myeloid leukemia (AML), the findings could be greatly affected by the cell type used in the experiment (e.g., in [*E-GEOD-12662, E-GEOD-14924, E-GEOD-35010*]). We therefore considered the cell type as one of the factors. We used a broad categorization of blood versus non-blood cell types.

### *The number of differentially expressed genes (pDEG)*

For each dataset, we performed a differential expression analysis by fitting a linear model for microarray data (well-known as limma) [45] and controlling the false discovery rate (FDR at 5%) defined as expected proportion of false rejection among the rejected hypotheses using the Benjamini and Hochberg (BH) procedure [46].

### *The within-class correlation level (withincor)*

We constructed the within-class correlation matrices for each dataset. A shrinkage approach was applied to estimate the correlation matrix to deal with the high dimensionality and sparsity [47]. We took the average of absolute pairwise correlations within each class and averaged those values over the two classes to represent the level of the within-class correlation coefficient for a dataset.

### *The fold change (fc)*

We calculated the fold change for each actual probeset as the absolute difference of the mean of log_2_ expressions between samples in two groups, divided by the pooled standard deviation. We summarized the fold changes in each dataset as the mean fold changes from all probesets.

## Random effects logistic regression

The nine classification models were built in the twenty five gene expression microarray datasets. We considered these datasets as clustered data, where the selected studies and the classification methods act as clusters. Further, in each study, we treated the accuracy as a grouped binomial variable, for which we had the number of samples that were correctly and incorrectly classified. We therefore evaluated the six aforementioned predictive factors for classification accuracy by a logistic random intercept regression model [48]. The logistic random effects model is the generalization of the linear mixed model to binomial outcomes. In this case, the sigmoid logistic link function is applied to the common linear mixed model and the error distribution is binomial instead of normal.

As the accuracy is well known to be biased towards the majority class, the random intercept logistic model was corrected by the class imbalance level, which was always included in the regression model. For the *l* ^*th*^ study factor, the random effects model is written as

$$ log\left(\frac{\pi \left({x}_{iSM}\right)}{1-\pi \left({x}_{iSM}\right)}\right)=\left({\beta}_0+{\vartheta}_{0s}+{\vartheta}_{0M}\right)+{\beta}_1 class\_ imbalanc{e}_S+{\beta}_2 predictive\_ facto{r}_{lS}, $$

where *π*(*x*_*isM*_) is the probability of a sample *i* in study *S* to be correctly classified with the classification model *M; ϑ*_0*S*_ and *ϑ*_0*M*_ are the random intercepts with respect to study *S* (*ϑ*_0*S*_ ~ *N*(0, *σ*_0*S*_^2^)); and classification method *M* (*ϑ*_0*M*_ ~ *N*(0, *σ*_0*M*_^2^))*.* All the aforementioned study factors were evaluated by simple and multiple logistic random intercept regression models. Multiple regression evaluation was done by a forward selection approach. In each step, two nested models, with and without a particular study factor, were compared by Akaike’s information criterion (AIC). Each factor *l* was also evaluated by its explained-variation of the random intercept variance term,

3 $$ va{r}_l=\frac{\sigma_{null}^2-{\sigma}_l^2}{\sigma_{null}^2}, $$

where *σ*_*null*_^2^ is the random intercept variance from a model with “class imbalance” only (referred to as null model). Since the logistic models have two random effects variables, *σ*_*null*_^2^ is the combined variance of the study (*σ*_0*S*_^2^) and the classification method (*σ*_0*M*_^2^) random effect from a null model. Meanwhile, *σ*_*l*_^2^ is the combined variance from a random effects model with the *l* ^*th*^ factor. The explained variation of all significant factors in the model (we refer to as “final model”) was also evaluated. It was calculated by replacing the *σ*_*l*_^2^ in Eq.3 with the combined variance in the final model.

We evaluated the stability of the simple and multiple random effect logistic regression models by performing Jackknife resampling analysis. In each iteration, one study was left out and the model building process was repeated using the retained studies.

## Software

All statistical analyses were performed in R software by using these following packages: *affy* for preprocessing procedures [49], *CMA* for predictive modeling [35], *limma* for fitting a linear model for microarray data [45], *lme4* for random effects linear model [50] and *ggplot2* for data visualization [51]. The R scripts are available in the Supplementary Material (Additional File 10).

## Additional file

Additional file 1:
**A brief description of the selected microarray gene expression experiments.**

Additional file 2:
**Table S1.** Stability analysis of univariable random effects logistic regression models via Jackknife resampling.
Additional file 3:
**Table S2.** Study factors that were included in the multivariable random effect logistic regression models via Jackknife resampling.
Additional file 4:
**Figure S1.** Boxplot of Disease type against the classification model accuracy.
Additional file 5:
**Figure S2.** Boxplot of Medical question against the classification model accuracy.
Additional file 6:
**Figure S3.** Boxplot of Cell Type against the classification model accuracy.
Additional file 7:
**Figure S4.** Plot of the Fold Change against the classification model accuracy.
Additional file 8:
**Figure S5.** Plot of the Number of Differentially Expressed Genes (in the log scale) against the classification model accuracy.
Additional file 9:
**Figure S6.** The visualization of the generated gene expression datasets with the scenario of fc1 = +,fc2 = +,cc1 = cc2 = −. Abbreviations: fc1(2): fold change of gene 1 (2); cc1(2): correlation coefficient of gene 1 (2).
Additional file 10:
**R script.**
