# Supplementary material for: Factors affecting the accuracy of a class prediction model in gene expression data
Source: BMC Bioinformatics. 2015 Jun 21;16:199. doi: 10.1186/s12859-015-0610-4 (PMC4475623; doi:10.1186/s12859-015-0610-4)
Supplement: Additional file 1: — A brief description of the selected microarray gene expression experiments. [file 12859_2015_610_MOESM1_ESM.pdf]

BRIEF DESCRIPTION OF SELECTED DATASETS

Ulcerative Colitis (UC1). A subset (cohort A) of data from two cohorts of patients who received their first treatment with infliximab for refractory ulcerative colitis. Pre-treatment colonic mucosal expression profiles were compared for responders (n=8) and non-responders (n=16) with the goal to identify mucosal gene signatures predictive of response to infliximab in patients with ulcerative colitis. (ArrayExpress ID: E-GEOD-14580)

Ulcerative Colitis (UC2). A prospective accrued cohort of 128 children between 2-18 years of age, hospitalized for intravenous corticosteroid therapy for acute ulcerative colitis that was all treated with methylprednisolone. Blood samples were collected on day 3 after admission to corticosteroid treatment and on day 5 of corticosteroid treatment, patients were determined to be non-responders if they needed a second line of medical therapy (e.g. infliximab) or colectomy. The corticosteroid-responsive (n=20) and corticosteroid-refractory (n=20) patients were randomly selected for analysis of mRNA expression with the goal to determine whether early initiation of intravenous corticosteroid treatment is associated with response. (ArrayExpress ID: E-GEOD-21231)

Ulcerative Colitis (UC3). A gene expression datasets were developed from twenty eight patients either with Chron's disease or ulcerative colitis. Total RNA obtained from intestinal biopsies were hybridized by Affymetrix GeneChip HG U133 Plus 2.0. We left out the healthy samples for our further analyses. (ArrayExpress ID: E-GEOD-36807)

Ulcerative Colitis (UC4). A placebo-controlled study of infliximab was conducted in total of forty eight ulcerative colitis patients. Samples' RNA were extracted and hybridized to be measured its gene expression in the baseline, 8-week and 30-week after receiving a treatment. The experiment was meant to compare the gene expression profiles from infliximab responders and non-responders, with respect to different dose and duration of treatment. We took gene expression data of responders (n=7) and non-responders (n=7) in the week 8 and received 5 mg/kg dose of infliximab. (ArrayExpress ID: E-GEOD-23597)

Ulcerative Colitis (UC5). The gene expression experiment was used to get more understanding of the natural history of Chron's disease and ulcerative colitis. Peripheral CD8 and CD4 T-cells were isolated from patients with active and untreated Chron's disease as well as patients with ulcerative colitis. The study found that the microarray gene expression data, particularly obtained from CD8 T-cells, improve the accuracy of diagnostic prediction in patients with Chron's disease and ulcerative colitis. We used 59

samples resulted from CD8 T-cells, in which 30 and 29 patients with ulcerative colitis and Chron's disease, respectively. (ArrayExpress ID: E-MTAB-331)

Ulcerative Colitis (UC6). The study was aimed to investigate the predictive diagnosis of pre-inflammatory state exists in biopsies of non-inflamed UC colon. Descending colon samples were taken from eight and nine UC samples with and without macroscopic signs of inflammation, respectively. The five healthy controls were also included in the gene expression experiment, but we did not incorporate those samples in to our study. (ArrayExpress ID: E-GEOD-9452)

Ulcerative Colitis (UC7). An underlying process of the ulcerative colitis and crohn's disease was unclear yet. A microarray gene expression experiment was conducted in patients who were fall into one of these disease states, Normal (n=4), Crohn's disease (n=19), ulcerative colitis (n=11), and other type of colitis (n=4). In order get binary classification case, we used gene expression data of patients with Crohn's disease and ulcerative colitis. (ArrayExpress ID: E-GEOD-6731)

Asthma (AST1). A subset (severe therapy-resistant and mild asthma) of data from a study in which the white blood cells of children with severe therapy-resistant asthma (n=17) and controlled or mild asthma (n=19) identified from a Swedish nation-wide study, together with recruited healthy controls (n=18) were profiled with the goal to identify global patterns of gene expression in severe therapy-resistant versus controlled asthma and healthy controls. We left out healthy controls to get binary class. (ArrayExpress ID: E-GEOD-27011)

Asthma (AST2). A cross-sectional study that included seventeen patients (6 allergic asthma and allergic rhinitis; 5 allergic rhinitis; and 6 healthy controls) was aimed to observed the differential responses of the upper and lower airway epithelium to double stranded, which lead to the presence of asthma and allergic rhinitis. The microarray was used to analyze extracted RNA from bronchial epithelial and nasal cells. For our analyses, we used gene expression data resulted from bronchial epithelial cell of patients with asthma and rhinitis before the treatment. (ArrayExpress ID: E-GEOD-51392)

Asthma (AST3). The gene expression experiment aimed to observe the changes of mRNA expression in non- (n=4) and severe asthma (n=8) patients as well as healthy controls (n=8). The CD8+ and CD4+ T cells were isolated and total RNA was extracted. (ArrayExpress ID: E-GEOD-31773)

Dystonia (DYS). The dataset is from a DYT1 dystonia (an autosomal-dominantly inherited movement disorder) study in which whole blood gene profiles of non- and manifesting carriers were compared. The gene expression datasets were obtained by hybridization of cDNA from whole blood samples from each patient. The training and testing datasets were combine and for further study we considered the 22

samples that belong to the carrier and 23 samples from symptomatic group. (ArrayExpress ID: E-GEOD-19419)

Human immunodeficiency virus (HIV1). Gene expression profiling experiments had been done in the three regions of the human brain, i.e. basal ganglia, white matter, and frontal cortex, to find implication of HIV and the neurological impairment and inflammation with HIV infection. Samples from the three brain regions were collected for RNA isolation in order to perform microarray analysis. We used gene expression datasets from the basal ganglia samples of the infected HIV patients. Further, we merged gene expression data from HIV-associated dementia and HIV encephalitis patients. The diagnostic prediction was based on the binary classes between HIV infected patients and HIV infected with complication. (ArrayExpress ID: E-GEOD-35864)

Human immunodeficiency virus (HIV2). As one of the further implications of gene expression profiling, this study is aimed to understand the resistance of individual with HIV infection for vaccine development. Patients were grouped into either HIV resistant or HIV susceptible. CD4+ T cells from peripheral blood mononuclear cells were taken from sex-workers in Nairobi, Kenya. The total RNA was isolated and hybridized in Affymetrix HG U133 Plus 2.0. (ArrayExpress ID: E-GEOD-14278)

Human immunodeficiency virus (HIV3). The peripheral blood mononuclear cells from twenty male donors (early HIV infection (n=5), chronic progressive HIV infection (n=5), non-progressive HIV infection (n=5) and healthy individuals (n=5)) were examined. The gene expression were measured by from CD4+ and CD8+ T cells of each donor. The study aimed to compare the expression profiles from various stage of HIV infected patients between CD4+ and CD8+ T cells. (ArrayExpress ID: E-GEOD-6740)

Psoriasis (PSO). Pre-treatment peripheral blood of moderate-to-severe psoriasis patients that were treated with Alefacept treatment and were classified at the end of the treatment either as non- (n=7) or responders (n=9). The goal is to use pre-treatment gene expression profiles to predict whether or not a patient will respond to Alefacept treatment. (ArrayExpress ID: E-GEOD-18948)

Kawasaki disease (KD). The Egami scoring system had been used to group patients with kawasaki disease into IVIG responsive (Group A, n=6) or IVIG resistant group (Group B, n=11) before starting the treatment. All patients in Group A received IVIG, meanwhile patients in Group B randomly assigned into IVIG (n=6) or IVIG+IVMP (n=5) treatment. A sample of 2.5 mL whole blood had been taken from each patient to isolate its total RNA. These samples had been used to determine the response status of patients.

In our study, we used resistance (n=6) and responsive (n=6) patients who had been treated with IVIG. (ArrayExpress ID: E-GEOD-16797)

Diabetes mellitus (dia1). The microarray gene expression had been used to profile the mRNA expression from people with type 2 diabetes (n=45), glucose intolerant (n=26), and normal samples (n=47). Total RNA had been extracted from skeletal muscle of the donors. RNA then hybridized in Affymetrix HG U133 Plus 2.0 chip. The datasets from normal samples were not included into further analyses. (ArrayExpress ID: E-GEOD-18732)

Diabetes mellitus (dia2). The DNA microarray had been used to identify a set of genes involved in oxidate phosphorylation those expression was coordinately decrease in human diabetic muscle. The experiment included male donors, in which further grouped into healthy control (n=17), donor with diabetes mellitus type 2 (n=18) and abnormal glucose tolerance (n=8). (ArrayExpress ID: E-CBIL-30)

***Alzheimer* (*ALZ1*).** Hippocampus tissue was taken from patients with Alzheimer's disease (22; 7 incipients, 8 moderate, and 7 severe) as well as control people (n=9). The diagnosis was based on the MiniMental Status Examination (MMSE) test. The re-classification of Alzheimer's patients was done to dichotomize the data, where we merged incipient and moderate patients, so that we have gene expression datasets from fifteen non-severe and seven severe patients. (ArrayExpress ID: E-GEOD-1297)

***Alzheimer* (*ALZ2*).** Microarray technology provide a molecular tool to understanding the etiology of neurodegenerative disease, in particular. The gene expression data had been obtained from medial temporal lobe of patients with Alzheimer's disease (n=7), Pick's disease (n=12), progressive supranuclear palsy (n=7) and normal samples (n=5). In order to get binary classification case, only samples from Alzheimer's and Pick's disease patients were included. (ArrayExpress ID: E-MEXP-2280)

Parkinson disease (PD). The differentially expressed genes in patients with neurodegenerative diseases and healthy controls were analyzed in this study. Whole blood samples had been taken from patients with Parkinson disease (n=50), neurodegenerative diseases other than PD (n=33), and healthy controls (n=23). The healthy samples were left out for our analysis. (ArrayExpress ID: E-GEOD-6613)

Heart failure (HF). A subset of data from a study in which microRNAs (miRNAs) and mRNA expression profiles of type 2 diabetic ischemic heart failure (D-HF, n=12), non-diabetic ischemic heart failure (ND-HF, n=7) patients and healthy controls (n=5) were compared in parallel with the goal to evaluate the impact of miRNA dysregulations and their potential pathogenetic roles. The control group was excluded so that all samples were in the heart failure disease state. (ArrayExpress ID: E-GEOD-26887)

Gaucher (GAU). A subset (Type I & III) of data from an experiment that had been conducted to provide insight into the unique pathogenesis of five Type I, five Type III Gaucher disease patients and four controls. Control and patient fibroblast cultures had been established from the full-thickness, skin biopsies obtained under IRB of the National Institute of Neurological Disorders and Stroke protocols and had been compared with the goal to find a gene signature that could be used to improve diagnostic accuracy and potential novel therapies for patients. Ten samples that had developed the disease were used for our study. (ArrayExpress ID: E-GEOD-21899)

Craniosynostosis syndrome (CS). The disease is a skull abnormality condition that might be caused by a gene mutation. The cRNA from each patient's skin had been used to compare the gene expression among three monogenic syndromes group (Apert (n=10), Munke (n=10), and Saethre-chotzen syndrome (n=10)) and control group (n=10). For further analyses, we took Apert and Munke sample patients. (ArrayExpress ID: E-MEXP-2236)

Cystic fibrosis (CF). A gene expression study had been conducted in nine patients with chronic rhinosinusitis and six patients with both chronic rhinosinuitis and cystic fibrosis. Transcription profiling had been also done in nine healthy controls, but we excluded those data for our further analyses. (ArrayExpress ID: E-GEOD-10406)
