# Supplementary material for: Factors affecting the accuracy of a class prediction model in gene expression data
Source: BMC Bioinformatics. 2015 Jun 21;16:199. doi: 10.1186/s12859-015-0610-4 (PMC4475623; doi:10.1186/s12859-015-0610-4)
Supplement: Additional file 2: — Table S1. Stability analysis of univariable random effects logistic regression models via Jackknife resampling. [file 12859_2015_610_MOESM2_ESM.pdf]

Table S1 - Stability analysis of univariable random effects logistic regression models via jackknife resampling

Dataset*	Cell type	Medical question	N	pDEG+	Fold change	Correlation level
uc1	0.328	0.226	0.070	<0.001	<0.001	0.327
uc2	0.449	0.593	0.085	<0.001	<0.001	0.498
uc3	0.319	0.299	0.070	<0.001	<0.001	0.338
uc4	0.214	0.383	0.048	<0.001	<0.001	0.236
uc5	0.259	0.348	0.029	<0.001	<0.001	0.326
uc6	0.491	0.422	0.084	<0.001	<0.001	0.255
uc7	0.313	0.299	0.069	<0.001	<0.001	0.367
asth1	0.349	0.269	0.072	<0.001	<0.001	0.371
asth2	0.300	0.286	0.061	<0.001	<0.001	0.352
asth3	0.024	0.295	0.102	<0.001	0.001	0.441
dys	0.451	0.151	0.101	<0.001	<0.001	0.402
hiv1	0.324	0.302	0.067	<0.001	<0.001	0.335
hiv2	0.271	0.210	0.075	<0.001	<0.001	0.298
hiv3	0.504	0.279	0.042	<0.001	<0.001	0.466
pso	0.259	0.202	0.075	<0.001	<0.001	0.397
kd	0.384	0.402	0.043	<0.001	<0.001	0.302
dia1	0.259	0.268	0.108	<0.001	<0.001	0.443
dia2	0.311	0.300	0.067	<0.001	<0.001	0.206
alz1	0.342	0.303	0.073	<0.001	<0.001	0.355
alz2	0.378	0.331	0.078	<0.001	<0.001	0.297
parki	0.447	0.254	0.099	<0.001	<0.001	0.186
hf	0.224	0.249	0.042	<0.001	<0.001	0.540
gau	0.483	0.417	0.099	<0.001	<0.001	0.264
cs	0.558	0.475	0.076	<0.001	<0.001	0.483
cf	0.279	0.275	0.055	<0.001	<0.001	0.363

Table S1 provides the P values of the study factors in the univariable evaluation of the random effect logistic regression models if a particular study was excluded in each jackknife sampling.

* The selected studies that were removed in each jackknife sampling. The abbreviation of the datasets is as described in the Additional File 1.

+ The number of differentially expressed genes.
