# Supplementary material for: Factors affecting the accuracy of a class prediction model in gene expression data
Source: BMC Bioinformatics. 2015 Jun 21;16:199. doi: 10.1186/s12859-015-0610-4 (PMC4475623; doi:10.1186/s12859-015-0610-4)
Supplement: Additional file 3: — Table S2. Study factors that were included in the multivariable random effect logistic regression models via Jackknife resampling. [file 12859_2015_610_MOESM3_ESM.pdf]

Table S2 - Study factors that were included in the multivariable random effect logistic regression models via jackknife resampling

Dataset*	Cell type	Medical question	N	pDEG+	Fold change	Correlation level
uc1	-	-	-	v	-	-
uc2	-	-	-	v	v	v
uc3	v	-	-	v	v	v
uc4	v	-	-	v	v	v
uc5	-	-	-	v	v	v
uc6	-	-	-	v	v	v
uc7	-	v	v	v	-	v
asth1	-	-	-	v	v	v
asth2	v	-	-	v	v	v
asth3	-	v	v	v	-	v
dys	-	-	-	v	v	v
hiv1	-	-	-	v	v	v
hiv2	-	v	v	v	-	v
hiv3	-	v	v	v	v	v
pso	-	-	-	v	v	v
kd	-	-	-	v	v	v
dia1	v	-	-	v	v	v
dia2	-	-	-	v	v	v
alz1	-	-	-	v	v	v
alz2	-	-	-	v	v	v
parki	-	v	v	v	-	v
hf	-	-	-	v	v	v
gau	-	-	v	v	-	v
cs	-	-	-	v	v	v
cf	-	-	-	v	v	v

Table S2 shows the study factors that were included in the random effect logistic regression models if a particular study was excluded in each jackknife sampling. The study factors that were included in the multivariable logistic regression model were indicated by “v”. Otherwise, it was presented by “-”.

* The selected studies that were removed in each jackknife sampling. The abbreviation of the datasets is as described in the Additional File 1.

+ The number of differentially expressed genes.
