# Supplementary figures and images for: Factors affecting the accuracy of a class prediction model in gene expression data
Source: BMC Bioinformatics. 2015 Jun 21;16:199. doi: 10.1186/s12859-015-0610-4 (PMC4475623; doi:10.1186/s12859-015-0610-4)

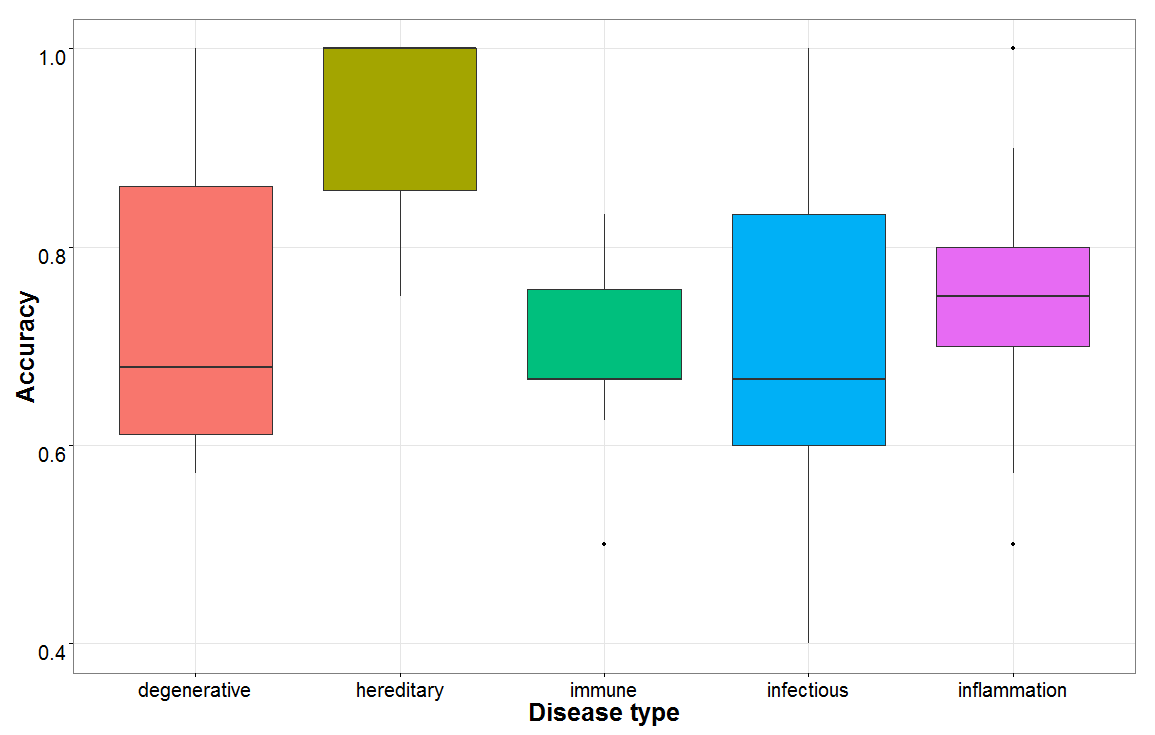

Supplement: Additional file 4: — Figure S1. Boxplot of Disease type against the classification model accuracy. [file 12859_2015_610_MOESM4_ESM.tiff]

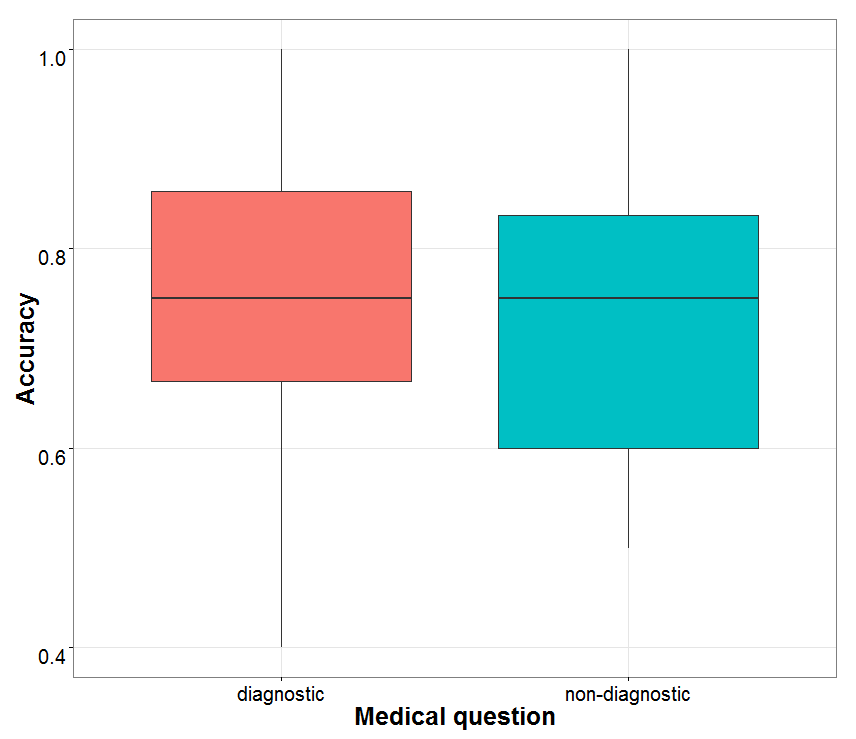

Supplement: Additional file 5: — Figure S2. Boxplot of Medical question against the classification model accuracy. [file 12859_2015_610_MOESM5_ESM.tiff]

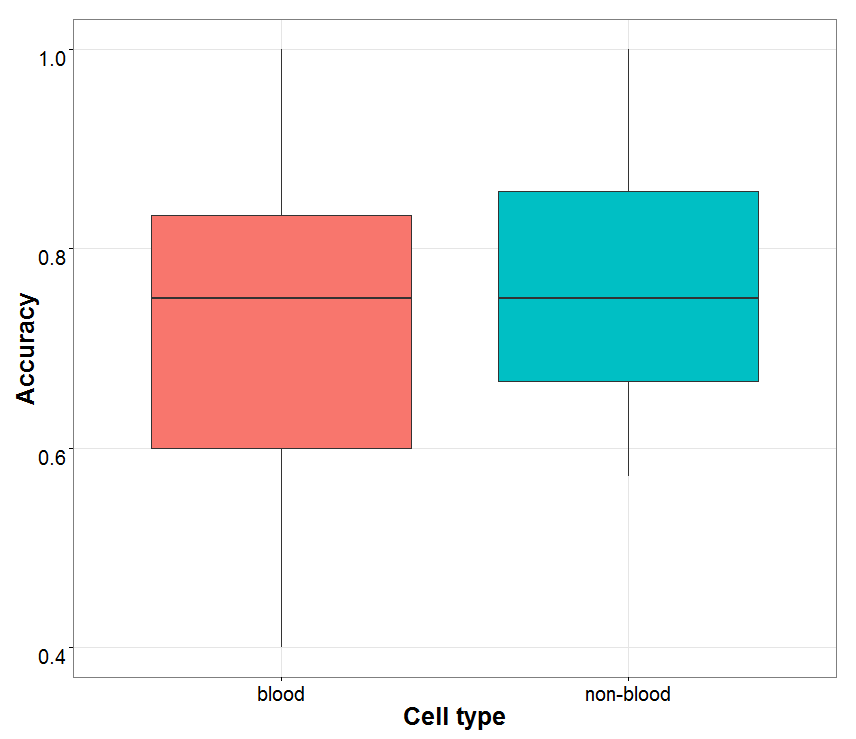

Supplement: Additional file 6: — Figure S3. Boxplot of Cell Type against the classification model accuracy. [file 12859_2015_610_MOESM6_ESM.tiff]

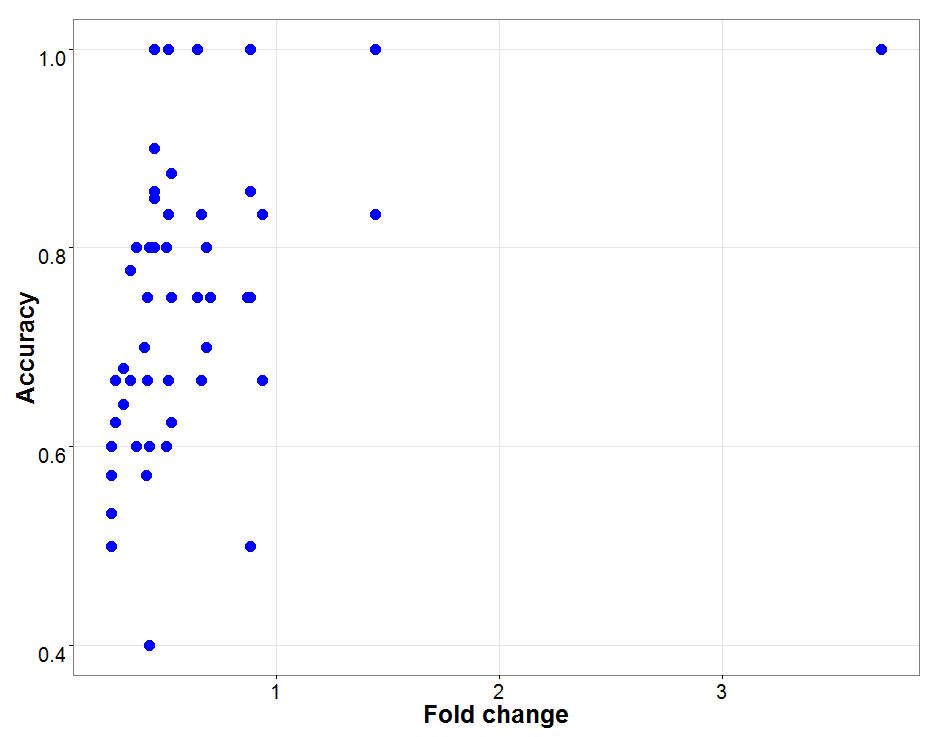

Supplement: Additional file 7: — Figure S4. Plot of the Fold Change against the classification model accuracy. [file 12859_2015_610_MOESM7_ESM.tiff]

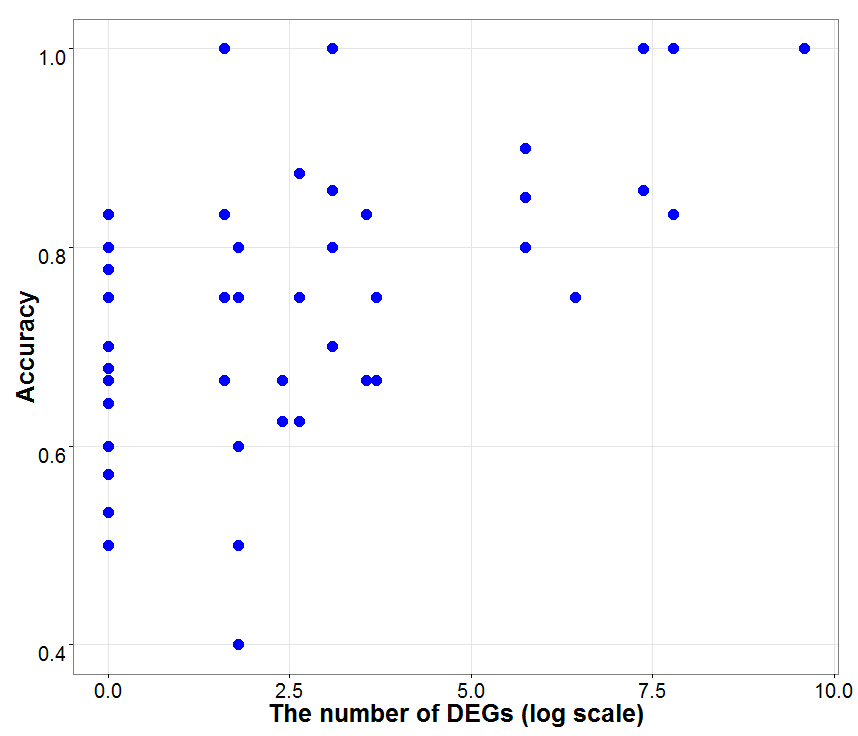

Supplement: Additional file 8: — Figure S5. Plot of the Number of Differentially Expressed Genes (in the log scale) against the classification model accuracy. [file 12859_2015_610_MOESM8_ESM.tiff]
