# Supplementary material for: Factors affecting the accuracy of a class prediction model in gene expression data
Source: BMC Bioinformatics. 2015 Jun 21;16:199. doi: 10.1186/s12859-015-0610-4 (PMC4475623; doi:10.1186/s12859-015-0610-4)
Supplement: Additional file 9: — Figure S6. The visualization of the generated gene expression datasets with the scenario of fc1 = +,fc2 = +,cc1 = cc2 = −. Abbreviations: fc1(2): fold change of gene 1 (2); cc1(2): correlation coefficient of gene 1 (2). [file 12859_2015_610_MOESM9_ESM.pdf]

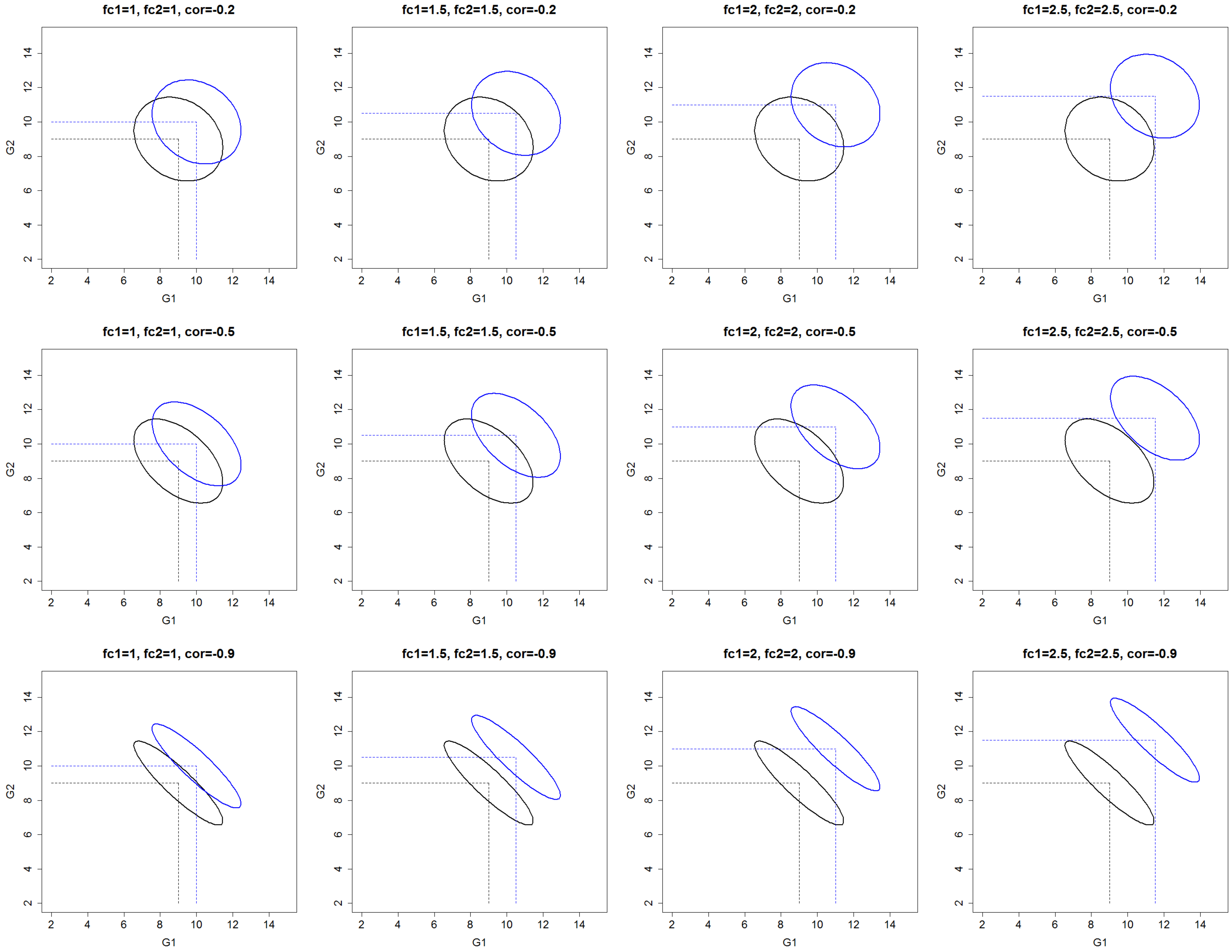
fc1=1, fc2=1, cor=-0.2
fc1=1.5, fc2=1.5, cor=-0.2
fc1=2, fc2=2, cor=-0.2
fc1=2.5, fc2=2.5, cor=-0.2
fc1=1, fc2=1, cor=-0.5
fc1=1.5, fc2=1.5, cor=-0.5
fc1=2, fc2=2, cor=-0.5
fc1=2.5, fc2=2.5, cor=-0.5
fc1=1, fc2=1, cor=-0.9
fc1=1.5, fc2=1.5, cor=-0.9
fc1=2, fc2=2, cor=-0.9
fc1=2.5, fc2=2.5, cor=-0.9
G1
G2
2
4
6
8
10
12
14
