# Supplementary material for: Factors affecting the accuracy of a class prediction model in gene expression data
Source: BMC Bioinformatics. 2015 Jun 21;16:199. doi: 10.1186/s12859-015-0610-4 (PMC4475623; doi:10.1186/s12859-015-0610-4)
Supplement: Additional file 10: — R script. [file 12859_2015_610_MOESM10_ESM.pdf]

# ADDITIONAL FILE 11. R SCRIPT

We provided R scripts to preprocessed raw datasets (PART 1), build classification models (PART 2) and evaluate the effect study factors to the performance of classification models by random effects regression model (PART 3). The detail description of each step can be found in the Method section.

## DOWNLOAD DATASET

We downloaded raw datasets from ArrayExpress online repository, with the accession ID as mentioned in the Table 1.

## PART 1. PREPROCESSIING

```
library(affy)
library(affydata)
library(AffyExpress)
library(genefilter)

# ================================================================================ #
# ================================= PREPROCESSING ================================ #
# ================================================================================ #
preprocess = function(affyversion){
  # Read CEL files, Normalization, background correction, and log2 transformation
  data.read = justRMA(normalize=TRUE, background=TRUE, bgversion=affyversion);
  datareadMat = as.matrix(data.read)

  # 1.At least 10% of sample (numChip=0.1*N) is greater than 5 (bg=5)
  t = 0.1*dim(data.read)[2]
  filter1 = as.matrix(Filter(data.read , numChip=t, bg=5))

  # 2. Remove genes whose std.dev < 0.5
  filter2 = filter1[which(apply(filter1,1,sd) > 0.5),]
  datafilter = filter2
  save(datafilter, file="datafilter.RData")
}
# ================================================================================ #

# affyversion = 1 : affymetrix version 1.0 - 1.0.2
# affyversion = 2 : affymetrix version >= 1.1
preprocess(affyversion=1)
```

# PART 2. PREDICTIVE MODELLING

We give an example of predictive modeling to classify severe(s) and non-severe(ns) patients in Alzheimer case (ArrayExpress ID: E-GEOD-1297). Predictive modeling for the other datasets could be done by using this following script to corresponding datasets of interest.

```
library(Biobase); library(limma); library(e1071); library(MASS);
library(plsgenomics); library(glmnet); library(pamr); library(mvtnorm);
library(MBESS); library(randomForest); library(CMA);


# ================================================================================ #
# =================== FUNCTIONS FOR DISCRIMINANT ANALYSIS METHODS =============== #
# ================================================================================ #
build.da = function(dataX, dataY, train, gene, ngene,dam.k){
  da.method = get(paste(dam.k,"CMA",sep=""))
  model.da = classification(X=dataX, y=dataY, learningsets=train, genesel=gene,
nbgene=ngene, classifier=da.method);
  acc = 1-summary(evaluation(model.da, measure="misclassification"))[4]
  return(acc)
}

test.discr= function(evalv.k,dam.k,topk,data,label,train,sel.train){
  ngene = topk[which((1-evalv.k)==min(1-evalv.k))[1]];
  acc = build.da(data, label, train, sel.train, ngene,dam.k)
  return(cbind(acc,ngene))
}
# ================================================================================ #


# ================================================================================ #
# ============================== DATA PREPARATION =============================== #
# ================================================================================ #
load("datafilter.RData")   #datafilter: preprocessed raw dataset, as resulted from PART 1
n = dim(datafilter)[2] ;p = dim(datafilter)[1] ;
group =
factor(c("s","ns","ns","s","s","s","ns","ns","ns","s","s",replicate(8,"ns"),"s","ns","ns"
))
# ================================================================================ #


# ================================================================================ #
# ==============================  MODEL BUILDING =============================== #
# ================================================================================ #
data = t(datafilter);
B=100 #the number of replications

#classification methods other than discriminant analyses
cm = c("svm","gbm","knn","rf","plr","scda","Lasso")
#discriminant analysis methods
dam = c("lda","dlda")

topK = c(5, 10, 15, 20, 25, 50, 55); nK = length(topK);
ACC=nc=nm=matrix(NA,length(cm),m);
```

```
topk = c(2,3,4,5);
ACC.da=nc.da=nm.da= matrix(NA,length(dam),m);

method = "limma";

for(i in 1:B){
  n.train = floor(2/3*length(label)); n.test = n-n.train
  train = GenerateLearningsets(y=label, method="MCCV",niter=1,
ntrain=n.train,strat=TRUE);
  sel.train = GeneSelection(X=data, y=label, learningsets=train, method=method);

  besttune.svm = besttune.gbm = besttune.knn = besttune.nnet = besttune.rf = besttune.plr
= besttune.scda = besttune.pls_rf = besttune.lda = besttune.fda = besttune.Lasso = list()

  cv.data = data[train@learnmatrix, ]
  cv.label = label[train@learnmatrix];
  cv.train = GenerateLearningsets(y=cv.label, method="LOOCV");
  cv.seltrain = GeneSelection(X=cv.data, y=cv.label, learningsets=cv.train,
method=method);

  # defining f-fold (LOOCV)
  F=numeric()
  for(f in 1:n.train){F[f]=length(which(cv.train@learnmatrix[f,] != 0))}
  fcv=min(F)

  # ---------------------------------------------------------------------- #
  # -------------------- 1. NON-DISCRIMINANT METHODS -------------------- #
  # ---------------------------------------------------------------------- #
  # TUNING PARAMETER(S)
  eval.v = matrix(NA,length(cm),nK);
  for(j in 1:nK){
    for(k in 1:(length(cm))){
      classif.method=get(paste(cm[k],"CMA",sep=""))
      tune.j=tune(X=cv.data, y=cv.label, learningsets=cv.train, genesel=cv.seltrain,
nbgene=topK[j], classifier=classif.method, fold=fcv);
      classif.validate = classification(X=cv.data, y=cv.label, learningsets=cv.train,
genesel=cv.seltrain, nbgene=topK[j], classifier=classif.method, tuneres=tune.j);
      eval.v[k,j] = summary(evaluation(classif.validate,
measure="misclassification"))[4];

      if(k==1){besttune.svm[[j]] = best(tune.j)}; if(k==2){besttune.gbm[[j]] =
best(tune.j)}
      if(k==3){besttune.knn[[j]] = best(tune.j)};
      if(k==4){besttune.rf[[j]] = best(tune.j)}; if(k==5){besttune.plr[[j]] =
best(tune.j)}
      if(k==6){besttune.scda[[j]] = best(tune.j)};
      if(k==7){besttune.Lasso[[j]] = best(tune.j)};
    }
  }
  # TESTING MODELS
  eval.t=matrix(NA,length(cm),m)
  for(k in 1:(length(cm))){
    idx.optgene = which(eval.v[k,]==min(eval.v[k,]))[1]
    classif.method=get(paste(cm[k],"CMA",sep=""))
    best.k=get(paste("besttune.",cm[k],sep=""))
    best.tune2 = as.matrix(unlist(best.k[[idx.optgene]]));
    param = unique(rownames(best.tune2)); best.param=matrix()
```

```
    for(x in 1:(length(param))){best.param[x] =
median(best.tune2[which(rownames(best.tune2)==param[x])])}
    p4 = topK[idx.optgene]

    if(k==1){classif.test = classification(X=data, y=label, learningsets=train,
genesel=sel.train, nbgene=p4, classifier=classif.method, cost=best.param)}
    if(k==2){classif.test = classification(X=data, y=label, learningsets=train,
genesel=sel.train, nbgene=p4, classifier=classif.method, n.trees=best.param)}
    if(k==3){classif.test = classification(X=data, y=label, learningsets=train,
genesel=sel.train, nbgene=p4, classifier=classif.method, k=best.param)}
    if(k==4){classif.test = classification(X=data, y=label, learningsets=train,
genesel=sel.train, nbgene=p4, classifier=classif.method, mtry=best.param[1],
nodesize=best.param[2])}
    if(k==5){classif.test = classification(X=data, y=label, learningsets=train,
genesel=sel.train, nbgene=p4, classifier=classif.method, lambda=as.numeric(best.param))}
    if(k==6){classif.test = classification(X=data, y=label, learningsets=train,
genesel=sel.train, nbgene=p4, classifier=classif.method, delta=as.numeric(best.param))}
    if(k==7){classif.test = classification(X=data, y=label, learningsets=train,
genesel=sel.train, nbgene=p4, classifier=classif.method,
norm.fraction=as.numeric(best.param))}

    acc = 1-summary(evaluation(classif.test, measure="misclassification"))[4];
    eval.t[k,i] = acc
    ACC[k,i] = acc;
    nc[k,i] = round(acc*n.test); #the number of correctly classified samples
    nm[k,i] = n.test - round(acc*n.test); #the number of incorrectly classified samples
  }

  # ------------------------------------------------------------------- #
  # ---------------------- 2. DISCRIMINANT METHODS ---------------------- #
  # ------------------------------------------------------------------- #
    # TUNING PARAMETER (OPTIMUM NUMBER OF GENES)
  evalv.da = matrix(NA,length(dam),length(topk));
  for(j in 1:length(topk)){
    for(k in 1:(length(dam))){
      evalv.da[k,j] = build.da(cv.data,cv.label,cv.train,cv.seltrain,topk[j],dam[k])
    }
  }

  # TESTING DISCRIMINANT METHODS
  acc.discr =  matrix(NA,length(dam),2)
  for(k in 1:(length(dam))){
    acc.discr= test.discr(evalv.da[k,],dam[k],topk,data,label,train,sel.train)
    acc.da= acc.discr[,1];
    ACC.da[k,i] = acc.da
    nc.da[k,i] = round(acc.da*n.test); #the number of correctly classified samples
    nm.da[k,i] =n.test-round(acc.da*n.test) #the number of incorrectly classified samples
  }

}
summaryall = list(rbind(ACC,acc.da),rbind(nc,nc.da),rbind(nm,nm.da))
# ==================================================================== #
```

## PART 3. RANDOM EFFECTS LOGISTICS REGRESSION

Once classification models were developed in all datasets, random effects logistic regression is used to evaluate the effect of selected study factors to the performance of classification models. The details of study factors are described in the Method Section and their values are depicted in the Table 1.

```
library(lme4)

# NC          : A vector of the number of correctly classified samples
# NM          : A vector of the number of miss-classified samples
# nprop       : A vector of class imbalance level

# ================================================================================== #
# ========================= UNIVARIATE EVALUATION ================================ #
# ================================================================================== #

modelnull = glmer(cbind(NC,NM)~  nprop + (1|disease) + (1|classif.method),
family=binomial)
summary(modelnull)

# 1. CELL TYPE
model1 = glmer(cbind(NC,NM)~  nprop + celltype + (1|disease) + (1|classif.method),
family=binomial)
summary(model1);

# 2. MEDICAL QUESTION
model1 = glmer(cbind(NC,NM)~  nprop + medicalquestion + (1|disease) + (1|classif.method),
family=binomial)
summary(model1);

# 3. SAMPLE SIZE
model1 = glmer(cbind(NC,NM)~  nprop + N + (1|disease) + (1|classif.method),
family=binomial)
summary(model1);

# 4. THE NUMBER OF DIFFERENTIALLY EXPRESSED GENES (LOG-TRANSFORMED)
log.ndeg = log(ndeg+1)
model1 = glmer(cbind(NC,NM)~  nprop + log.ndeg + (1|disease) + (1|classif.method),
family=binomial)
summary(model1);

# 5. FOLD CHANGE
model1 = glmer(cbind(NC,NM)~  nprop + fc + (1|disease) + (1|classif.method),
family=binomial)
summary(model1);

# 6. WITHIN CLASS CORRELATION COEFICIENT
model1 = glmer(cbind(NC,NM)~  nprop + cc + (1|disease) + (1|classif.method),
family=binomial)
summary(model1);

# ================================================================================== #
```

```
# ============================================================================== #
# ========================= MULTIVARIABLE EVALUATION ============================= #
# ============================================================================== #

model2 = glmer(cbind(NC,NM)~  nprop + log.ndeg + (1|disease) + (1|classif.method),
family=binomial)
summary(model2);

model2 = glmer(cbind(NC,NM)~  nprop + log.ndeg + fc2 +(1|disease) + (1|classif.method),
family=binomial);
summary(model2);

model2 = glmer(cbind(NC,NM)~  nprop + log.ndeg + fc2 + ccs_within + (1|disease) +
(1|classif.method), family=binomial);
summary(model2);

# This is a straightforward R script to evaluate the study factors with multiple
# regression random effects models by forward approach.
# The script shows the order of study factors (variables) that enter the model
# Detail description about the forward approach to the random effect multiple regression
# evaluation, is provided in the Method Section ("Random effect logistic regression"
# subsection)

# ============================================================================== #
```
